# CoCoNet—boosting RNA contact prediction by convolutional neural networks

**DOI:** 10.1093/nar/gkab1144

**Published:** 2021-12-06

**Authors:** Mehari B Zerihun, Fabrizio Pucci, Alexander Schug

**Affiliations:** John von Neumann Institute for Computing, Jülich Supercomputing Centre, Forschungszentrum Jülich, 52428 Jülich, Germany; Steinbuch Centre for Computing, Karlsruhe Institute of Technology, 76344 Eggenstein-Leopoldshafen, Germany; John von Neumann Institute for Computing, Jülich Supercomputing Centre, Forschungszentrum Jülich, 52428 Jülich, Germany; Computational Biology and Bioinformatics, Université Libre de Bruxelles 1050, Brussels, Belgium; John von Neumann Institute for Computing, Jülich Supercomputing Centre, Forschungszentrum Jülich, 52428 Jülich, Germany; Faculty of Biology, University of Duisburg-Essen, 45117 Essen, Germany

## Abstract

Co-evolutionary models such as direct coupling analysis (DCA) in combination with machine learning (ML) techniques based on deep neural networks are able to predict accurate protein contact or distance maps. Such information can be used as constraints in structure prediction and massively increase prediction accuracy. Unfortunately, the same ML methods cannot readily be applied to RNA as they rely on large structural datasets only available for proteins. Here, we demonstrate how the available smaller data for RNA can be used to improve prediction of RNA contact maps. We introduce an algorithm called **CoCoNet** that is based on a combination of a Coevolutionary model and a shallow Convolutional Neural Network. Despite its simplicity and the small number of trained parameters, the method boosts the positive predictive value (PPV) of predicted contacts by about 70% with respect to DCA as tested by cross-validation of about eighty RNA structures. However, the direct inclusion of the CoCoNet contacts in 3D modeling tools does not result in a proportional increase of the 3D RNA structure prediction accuracy. Therefore, we suggest that the field develops, in addition to contact PPV, metrics which estimate the expected impact for 3D structure modeling tools better. CoCoNet is freely available and can be found at https://github.com/KIT-MBS/coconet.

## INTRODUCTION

Ribonucleic acid (RNA) is one of biomolecular key players in cells and plays significant roles in many biological activities such as the coding, regulation and expressions of genes. For example, non-coding RNA is involved in genetic regulation acting on transcriptional and translational machinery (1,2) thus enables life as we know it. Since RNA function is closely related to its three-dimensional (3D) structure, experimental techniques such as X-ray diffraction and nuclear magnetic resonance (NMR) are the methods of choice to experimentally determine RNA 3D structure. However, these approaches can be very challenging for RNA often characterized by a high conformational flexibility. This is reflected in the limited number of RNA 3D structures in the Protein Data Bank (PDB) representing only few percents of the total number of all PDB entries (3). The large majority of known RNAs remain thus still structurally unresolved and is sometimes even called the dark matter of the biomolecular universe (4).

Computational methods can be a powerful tools to complement experimental efforts by predicting and analyzing RNA structures. Also called *in-silico* tools they can be used alone or in combination with experimental and statistical methods. When direct structure determination is not feasible, indirect measurement might still be possible. To improve the interpretation of such indirect experimental data, this data can be integrated in computational modeling tools. For instance, small angle X-ray scattering (SAXS), and single molecule Förster Resonance Energy Transfer (FRET) data have been fruitfully used in combination with molecular dynamics simulations of proteins (5,6). Similarly, homology modeling, fragment- and physics-based structure prediction approaches have been developed in the last decade (4,7–15) and their accuracy and efficiency, while remaining limited especially for large RNAs, is constantly improving as shown in the four blind prediction experiments RNAPuzzle (16–19).

Likewise, information about spatial proximity of nucleotides inferred by statistical approaches from multiple sequence alignment (MSA) of RNA families can be utilized as spatial constrains in molecular modeling tools (4,20–22). Since structure prediction methods in tandem with these prior information have shown to be more accurate than used alone, these statistical methods have received lots of attention. A wide range of methods based on different implementations of direct coupling analysis (DCA) (23,24) of coevolving nucleotides, including the mean-field approximation, pseudo-likelihood maximization, sparse inverse covariance estimation and Boltzmann learning (25–31), have thus been recently introduced to improve the reliability of predicting nucleotide pairs in spatial proximity. In particular, DCA can distinguish correlations resulting from direct or indirect effects of nucleotide interactions, which strongly increases its prediction accuracy of contacts compared to old methods such as Mutual Information (MI).

To evaluate the performance of DCA-based methods on RNA contact prediction, we compared different methods on a well curated dataset of RNA structures (32). In the analysis we did not observe any significant variation of predicted contact accuracy among the algorithms. In particular and in contrasts to results for proteins, we did not detect significant accuracy differences between mean-field and pseudo-likelihood maximization. Quite recently machine learning-based approaches have shown to astonishingly improve the prediction of protein contact maps and to considerably boost the protein 3D structure prediction (33–35). These methods rely on the ability of deep neural networks to identify patterns in the input data using multiple levels of abstraction and have been already used to dramatically improve fields such as the computer vision and speech recognition (36,37).

These approaches, however, are characterized by a huge number of free parameters and require big datasets of 3D structures for their training and thus cannot be easily extended to RNA structure prediction due to the limited number of available experimentally resolved structures. Here, we thus focus not on deep but on shallow Neural Networks. In particular, we construct our approach CoCoNet as combination of the mean-field DCA approach with a shallow Convolutional Neural Network. We will demonstrate the approach’s ability to improve RNA contact prediction, while keeping the number of free parameters to train the network limited to assure the generalization of its performance.

## MATERIALS AND METHODS

### Coevolution models

Mutations play an essential role in shaping the evolution of all biomolecules. Their large majority have a neutral or, more often, deleterious effect on biomolecular fitness. Only few mutations lead to new functions. Evolutionary pressure acts on the biomolecules to counteract deleterious effects and restore their functional states favoring secondary compensatory mutations. The interactions between these mutations can be traced in the biomolecule’s evolution and be observed in multiple sequence alignments (MSAs) of homologous proteins or RNA. A series of co-evolutionary methods have been developed to capture the sequence variability in MSAs such as the Direct coupling analysis (DCA) (23–25). DCA is an inverse statistical method that is able to identified pairs of residues that co-evolved during evolutionary history and thus are likely to be in spatial adjacency in the three-dimensional structure of a protein/RNA molecule.

Let consider a sequence of nucleotide bases σ = *a*_1_*a*_2_*a*_3_...*a*_*L*_ of length *L* containing residues or a gap at sites 1, 2, 3, ..., *L*. The probability *P* of observing this sequence in a MSA is given by the following expression:

(1) $$\begin{equation*} P(\sigma ) = \frac{1}{\mathcal {Z}}\exp \left(\sum _{i=1}^{L-1}\sum _{j=i+1}^{L} J_{ij}(a_i, a_j) + \sum _{i=1}^{L}h_i(a_i) \right), \end{equation*}$$

where $\mathcal {Z}$ is the normalization constant (also known as partition function); *J*_*ij*_(*a*_*i*_, *a*_*j*_) are the couplings and *h*_*i*_(*a*_*i*_) are local fields. Finding a solution for equation 1 is computationally costly since the partition function scales as $\mathcal {O}(q^L)$. As a consequence, most algorithms of DCA rely on approximations. One of the most popular DCA algorithms is the mean-field direct-coupling analysis (mfDCA) (25), which shows good results for RNA (20). It is at the same time an accurate and fast method. As numerically more complex methods such as plmDCA (38)do not lead, unlike for proteins, to improvements for RNA contact prediction (4,20,21) we will here focus on mfDCA.

In mfDCA, the couplings are computed from the inverse of the empirical correlation matrix obtained from the MSA. Let *f*_*i*_(*a*_*i*_) be single-site frequency counts of the MSA for column *i* when occupied by a nucleotide/gap *a*_*i*_, and *f*_*ij*_(*a*_*i*_, *a*_*j*_) be the pair-site frequency counts for columns *i* and *j* when occupied by nucleotide/gap *a*_*i*_ and *a*_*j*_, respectively. These quantities are computed from the MSA as

(2) $$\begin{equation*} f_i(a_i) = \frac{1}{M_{eff} + \lambda }\left( \frac{\lambda }{q} + \sum _{m=1}^{M} \omega _m \delta _{a_i, a_i^{m}} \right) \end{equation*}$$

and

(3) $$\begin{equation*} f_{ij}(a_i, a_j) = \frac{1}{M_{eff} + \lambda } \left( \frac{\lambda }{q^2} + \sum _{m=1}^{M} \omega _m \delta _{a_i, a_i^m}\delta _{a_j, a_j^m} \right) \end{equation*}$$

where λ is the pseudocount for regularizing frequency counts; ω_*m*_ is weight of sequence *m* which is defined as the reciprocal of the number of similar sequences for a particular sequence similarity threshold; and *M*_*eff*_ is the effective number of sequences which is the sum of sequence weights. The correlation matrix *C* has elements *C*_*ij*_ = *f*_*ij*_(*a*_*i*_, *a*_*j*_) − *f*_*i*_(*a*_*i*_)*f*_*j*_(*a*_*j*_). The couplings of the model are obtained from

(4) $$\begin{equation*} J_{ij}(a_i, a_j) = -(C^{-1})_{ij}(a_i, a_j) \end{equation*}$$

for distinct site pairs *i* and *j*. The nucleic acid pairs are scored using the direct-information that is given by

(5) $$\begin{equation*} \mathcal {DI}_{ij} = \sum _a \sum _b p^{dir}_{ij}(a, b) \log \frac{p^{dir}_{ij}(a, b)}{f_i(a)f_j(b)}, \end{equation*}$$

where $p^{dir}_{ij}(a, b)$ is the direct probability defined by

(6) $$\begin{equation*} p^{dir}_{ij}(a, b) = \frac{1}{\mathcal {Z}_{ij}} \exp \left(J_{ij}(a, b) + \tilde{h_i}(a) + \tilde{h_j}(b)\right). \end{equation*}$$

and where parameters ($\tilde{h}_i$s) in equation 6 are obtained by requiring the direct probability marginals to be consistent with the single-site frequencies of the MSA. $\mathcal {Z}_{ij}$ is the normalization constant for $p^{dir}_{ij}(a, b)$. According to their DI scores, the pairs are then ranked. High-ranking pairs correspond to strongly coevolving nucleobases and thus tend to be in physical contact in the 3D structure of the RNA molecule (true positive/ TP prediction). However, lower ranking pairs are less likely to be a real or true positive contact (TP) and more likely to be a false positive prediction (FP) not in contact in the 3D structure. It should be noted that there is no hard threshold for the DI scores, e.g. a value above which TP rates are high and FP rates low. Instead, there is a gradual overall increase of FP as one goes down the ranked pairs. Also, it should be noted that coevolution can result not only from a single native conformation but also from multiple conformations, i.e. FP can be TP in other contexts. Examples include active and inactive conformations (39) or competition of inter- and intra-contacts in homodimers (40).

### Convolutional neural networks

Convolutional neural networks (CNNs) have been extensively used in the last decades in a wide range of applications that range from accurate learning of patterns in images to speech recognition (36,37,41). The success of CNNs resides in their ability to identify patterns in the input data using multiple levels of abstraction through a hierarchy of different layers of convolution. These artificial networks are composed by three kinds of layers in addition to the input and output layers. The first one is the convolution layer that applies a convolution operation on the input layer, the second ones are the pooling layers that perform downsampling operations and finally there are the fully connected layers whereby neurons are connected with all neurons in the preceding layers.

The tremendous effort devoted to the improvements of CNN architectures aims to make CNN scalable to larger and increasingly complex systems. Indeed from the simple LeNet architecture introduced in (42) consisting of three convolution, two pooling and a fully connected layers, a series of deeper CNNs that show improved performances such as AlexNet (43), ZFNet (44), GoogleNet (45) and VGGNet (46), have been introduced in the literature.

The increased level of complexity of these networks is reflected in the number of free parameters to train that range from 60k for LeNet to about 1380k for VGGNet. However, despite the accurate performances of these networks, this huge number of parameters makes the training slow and limits generalization (47).

When the training dataset is very small as for a RNA structure dataset(48), the deep network approach has to be completely ruled out to avoid overfitting and to allow reasonable generalization. For all these reasons we thus chose to employ a shallow convolutional neural network covered in the next subsection. Indeed these type of CNNs (49,50), that have just from one to few hidden convolutional layers, while keeping good performances, are characterized by a low training time and a reduced number of free parameters.

### Convolution on coevolution

**Figure 1.**
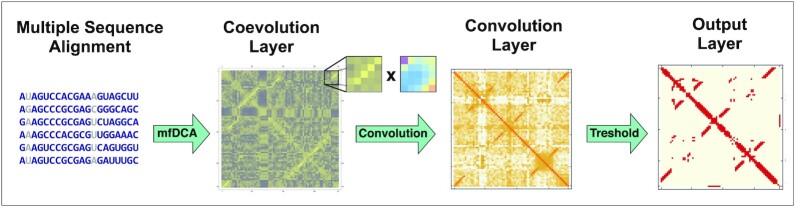
Schematic workflow of CoCoNet architecture with all different layers.

In order to improve contact prediction accuracy from RNA multiple sequence alignments, we here design a method called CoCoNet that is based on a combination of DCA with convolutional neural network approaches. CoCoNet is motivated by the simple observation that contact maps of RNA are not random but instead show ordered patterns of contacts. It’s very likely that nucleotide pairs close to other pairs that are in physical contact are also true contacts themselves. CNNs are a systematic method to identify patterns from DCA contact map prediction and filter out noisy and unwanted artifacts. The architecture of our CoCoNet method is schematically depicted in Figure 1 and is constituted by different layers.

- The input layer is simply given by the MSA of the target RNA sequence of length *L* with its homologous. Note that MSA are trimmed by selecting only positions corresponding to the specific RNA target sequence.
- The first layer is the coevolutionary layer. In this layer the DCA scores of nucleotide-nucleotide pairs are computed using a mean-field DCA approach. This step is performed using the mean-field algorithm implementation in pydca (30). A 2D map of size *L* × *L* is then constructed from these DCA scores assigning to each (*i*, *j*) pair of the target sequence the corresponding DCA score.
- The second layer is the convolutional layer. As a first step we perform a padding operation of size *p* = (*d* − 1)/2. Then a *d* × *d* filter matrix (with *d* chosen here to be equal to 3, 5 and 7) is used to perform convolution across the 2D DCA contact map obtained from the previous padded layer. This results in a new 2D contact map of size *L* in which each entry corresponds to a sort of re-weighted DCA scores.
- The output layer consist in selecting the *n* pairs from the previous layer map having the *n* highest re-weighted DCA scores and consider them as true contacts while giving a vanishing score for all the others. The choice of the number of contacts *n* that leads to reliable predictions is discussed in the results section. It depends on the system size as well as on the contact definition.

### The dataset of RNA structures

In order to train CoCoNet, we have to select a dataset of RNA structures. Here, we chose the well-curated dataset presented in (48) in which there are about seventy RNA structures of high resolution and their corresponding RFAM family of homologous RNA (51). We select a subset $\mathcal {S}$ of 57 entries associated to unique families in the RFAM database and discard similar structures that belong to the same family to avoid any bias at the training state. $\mathcal {S}$ is further divided in two subsets called $\mathcal {S}^{H}$ and $\mathcal {S}^{L}$ containing all entries associated to RFAM with *M*_*eff*_ greater than, and less than or equal to 70.0. Indeed nucleotide contact prediction performance depend on *M*_*eff*_ (48) and only predictions for RFAM family with *M*_*eff*_ larger than about 50–100 provide highly reliable results. The $\mathcal {S}^{H}$ and $\mathcal {S}^{L}$ contains 28 and 29 RFAM families respectively.

We have also constructed an additional independent test set of RNA structures by relaxing the structure resolution criteria used to construct $\mathcal {S}$ (48). In particular, here, we also consider NMR structure and crystal structure with a resolution higher than 3.6 Å. In this way, we have collected 23 additional RNA structures in a set called $\mathcal {T}$ that similarly to the dataset $\mathcal {S}$ is then split into two subsets $\mathcal {T}^{H}$ an $\mathcal {T}^{L}$ associated to RFAM families with *M*_*eff*_ greater than and less than or equal to 70.0, respectively.

For each RFAM family, all collected sequences are then aligned using Infernal (52) and the corresponding covariance model (CM). CM is a specific profile stochastic context-free grammar used to score the alignment through a combination of sequences and RNA secondary structure consensus. For more technical details about this step, we refer the reader to (48) where multiple alignment tools and parameters have been tested in preparing MSAs.

All structures have been download from the Protein Data Bank (53) and annotations of the secondary structure have been computed using DSSR (54,55). The PDB structures used in this paper, the corresponding RFAM families, and their annotations are listed in Supplementary Table S1 and S2 and can be found in the GitHub repository https://github.com/KIT-MBS/coconet.

### Learning the filter matrix

To learn the filter matrix of CoCoNet we use a simple gradient backpropagation algorithm as our network contains a single convolutional layer without any nonlinear activation functions implying the objective function being convex. The limited size of the dataset allows processing the training data in a single batch. Basically, we compare the weighed contact maps for all the target sequences in our dataset that are obtained from MSAs via the coevolution plus convolutional layer with the real contact maps obtained from the PDB structures. Two nucleotides are considered as contacts in the structures if they have a pair of heavy atoms (i.e. non-hydrogen) that are less than 10 Å apart. For nucleotide pairs fulfilling this condition they are assigned a value of one in the real contact map, zero otherwise.

Given a target RNA sequence *R* belonging to the training dataset, we can define a function

(7) $$\begin{equation*} \mathcal {F}^{R}_{ij} = \left(\mathcal {W} \ast \mathcal {D}^{R}_{ij} - \delta (\mathcal {C}^{R}_{ij})\right)^{2}, \end{equation*}$$

where * is the convolution operation between $\mathcal {W}$ and $\mathcal {D}_{ij}$ that are the filter matrix and the local *d* × *d* $\mathcal {DI}$$\mathcal {DI}$ scores matrix (eq. 5) centered at residue pairs (*i*, *j*), respectively. The delta function $\delta (\mathcal {C}^{R}_{ij})$ is one when nucleotide *i* and *j* are in physical contact in the PDB structure and zero otherwise.

The convolution operation can in principle be done using several filter matrices. To limit the number of free parameters, CoCoNet is designed to use a maximum of two filter matrices. Their total number range from 9, for a single 3 × 3 filter matrix up to 98 for two 7 × 7 filter matrices. When two filter matrices are used, one of them performs convolution with Watson–Crick nucleotide pairs and the other on non-Watson–Crick pairs. More in detail, the modified convolution occurs between the WC-filter and the $\mathcal {D}_{ij}$ matrix in which only the *i*, *j* entries corresponding to (A,U) and (C,G) contacts are considered while the remaining entries of $\mathcal {D}_{ij}$ are set to zero. Vice-versa, the convolution for non-WC filters occur between the non-WC filter and a $\mathcal {D}_{ij}$ matrix in which only the contacts different from (A,U) and (C,G) are considered. The final result is then obtained as the sum of the convolution of the two filters.

The total cost function is then defined as

(8) $$\begin{equation*} \mathcal {F} = \sum _R\sum _{j>i+4} \mathcal {F}^{R}_{ij}, \end{equation*}$$

where the summation over *R* represents the summation over all the entries in the training dataset and that of *i* and *j* over all nucleotide pairs that are separated at least four nucleobases in the sequence of *R*. The cost function is minimized using Limited-memory BFGS algorithm using a standard implementation in Python’s Scipy library (56). To avoid overfitting in the training processes, the computation is done using a strict five-fold cross-validation in which the entire set $\mathcal {S}$ is first randomly partitioned in five parts, and then each part is used in turn as a test set by removing it from the dataset, training the network weights on the remaining four parts and used the so-trained model on the test part. To ensure the robustness of our results, this cross-validation procedure is repeated ten times by performing each time a random splitting of the entries in five-folds. Final results are then obtained as the average over all the set of cross-validated results.

### Modeling RNA 3D structure

To evaluate how much the CoCoNet contact prediction method impacts the 3D structure modeling of RNA molecules, we randomly chose a set of 10 RNA families (listed in Section 5 of the supplementary materials). Four of them belong to the $\mathcal {S}^{L}$ set corresponding to RFAM families with low *M*_*eff*_ number and the other ones to $\mathcal {S}^{H}$ that instead correspond to RFAM with higher values of *M*_*eff*_.

For the modeling of the 3D structure, we use the tool SimRNA (9) that is based on a Monte-Carlo-based approach for the sampling of the RNA conformational space and a statistical potential to evaluate the energy of the configuration. We repeat each simulation at least ten times using 20 replicas for each simulation. Further details on the simulation time and parameters can be found in Section 5 of the Supplementary Materials.

From all generated RNA conformations, we selected the 1% with lowest energy and clustered them. We then selected twenty clusters and computed their averaged RMSD value with respect to the experimental structure.

## RESULTS

### Coevolutional structural features

Here, we analyze the structural patterns observed in the coevolutional layer of our network since their understanding provides insight on how CoCoNet is able to identify them and enhance nucleotide–nucleotide contact prediction. In particular, we study these structural features by investigating the average DCA scores in a 7 × 7 window around nucleotide pairs following a similar approach to the one employed in (57) for proteins. In addition, numerical values of the CoCoNet filters can be found in Supplementary Figure S5 in the supplementary material.

**Figure 2.**
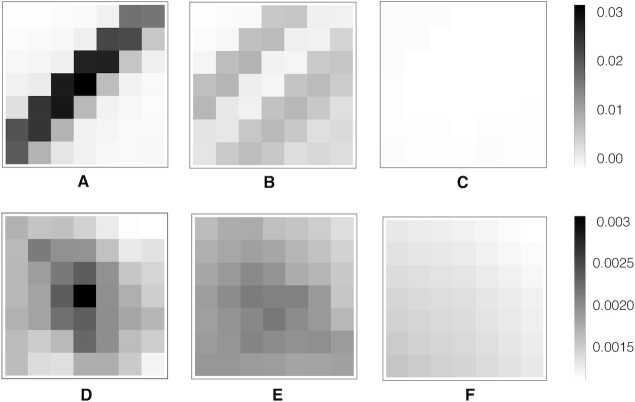
Structural features observed in the 2D coevolutionary map. Average DCA scores in a 7 × 7 window around all nucleobase pairs separated by a distance *r* ≤ 4 Å (**A**), 4 < *r* ≤ 10 Å (**B**) and *r* > 10 Å (**C**). Here, the intensity is proportional to the averaged DCA score of the corresponding element using the same color scale for (A), (B) and (C). In (**D–F**), we displayed the average DCA scores in a 7 × 7 window around all 3D nucleotide pairs separated according to the same criteria *r* ≤ 4 Å, 4 < *r* ≤ 10 Å and *r* > 10 Å, respectively. Here, the intensity color-scale is rescaled by a factor of about 10 when compared with (A), (B) and (C) to highlight the patterns.

In Figure 2A–C we plot this average for all type of contacts according to the spatial distance *r* between the closest heavy atoms (i.e. non-hydrogen) of a nucleotide pairs. At short distance (*r* ≤ 4 Å, Figure 2 A) we clearly observe a signal corresponding to a stem structure. For this pattern the coevolutionary scores are strongly reflecting the selection pressure of maintaining the corresponding secondary structure. At intermediate distance ( 4 < *r* ≤ 10 Å, Figure 2B) the observed patterns are weaker and essentially are dominated by stems pairs that are in the surrounding of the target contact. Finally at distance larger than 10 Å, there is essentially no signals as we can see in Figure 2C.

A similar pattern analysis shows when considering only nucleotide pairs that are far away from any secondary contacts, i.e. are outside a 9 × 9 window centered at any 2D contact. These patterns are shown in Figures 2D, E and F for distance *r* ≤ 4.0 Å, 4.0 < *r* < 10.0 Å and *r* > 10.0 Å, respectively. The first thing that we note from them is that coevolutionary signals from 3D contacts are much weaker than 2D ones: they are suppressed by a factor of about ∼10–20 and thus their intensity has been re-scaled accordingly to make them visible in Figure 2E–F. The patterns that we observe at short distances (2 D) has relatively stronger signals at the centre of the windows where the 3D contact is located and tends to decrease as we move away from the center. A somewhat similar signal with a center region characterized by a stronger coevolution can be observed also at intermediate distance (Figure 2E) even if the intensity is weaker and the pattern can be confused with the background without a further intensity rescaling (data not shown). Finally, at large distances (Figure 2F) no coevolutionary signals can be identified as expected.

### Contact prediction accuracy

Next, we test the accuracy of our contact prediction method as a function of some neural network characteristics such as the size of the filter matrices and its architecture. We use the CoCoNet prediction scores to rank nucleotide pairs since pairs showing high scores are likely to be spatially adjacent in the three dimensional structure of an RNA molecule. To assess CoCoNet’s performance, we computed its positive predictive value (PPV) that is the widely used metric for contact prediction. In addition, we also have evaluated CoCoNet's performance using the Mathews correlation coefficient (MCC) (see Supplementary Figures S3 and S4 of the Supplementary Materials).

**Figure 3.**
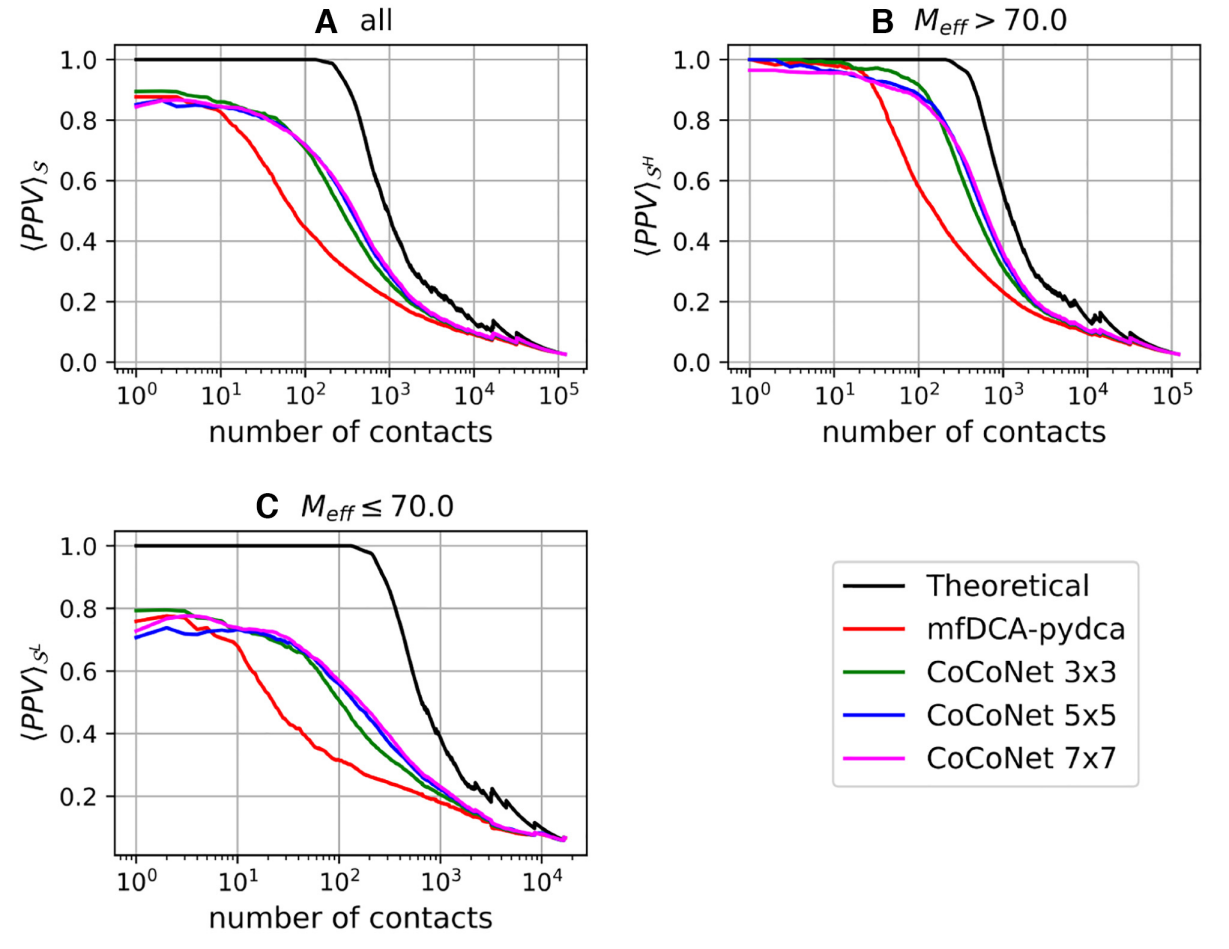
Average positive predicted value as a function of the number of ranked contacts for all RFAM families in the $\mathcal {S}$ (**A**), $\mathcal {S}^{H}$ (**B**) and $\mathcal {S}^{L}$ (**C**) datasets. CoCoNet’s results are obtained by 5-fold cross-validation.

**Figure 4.**
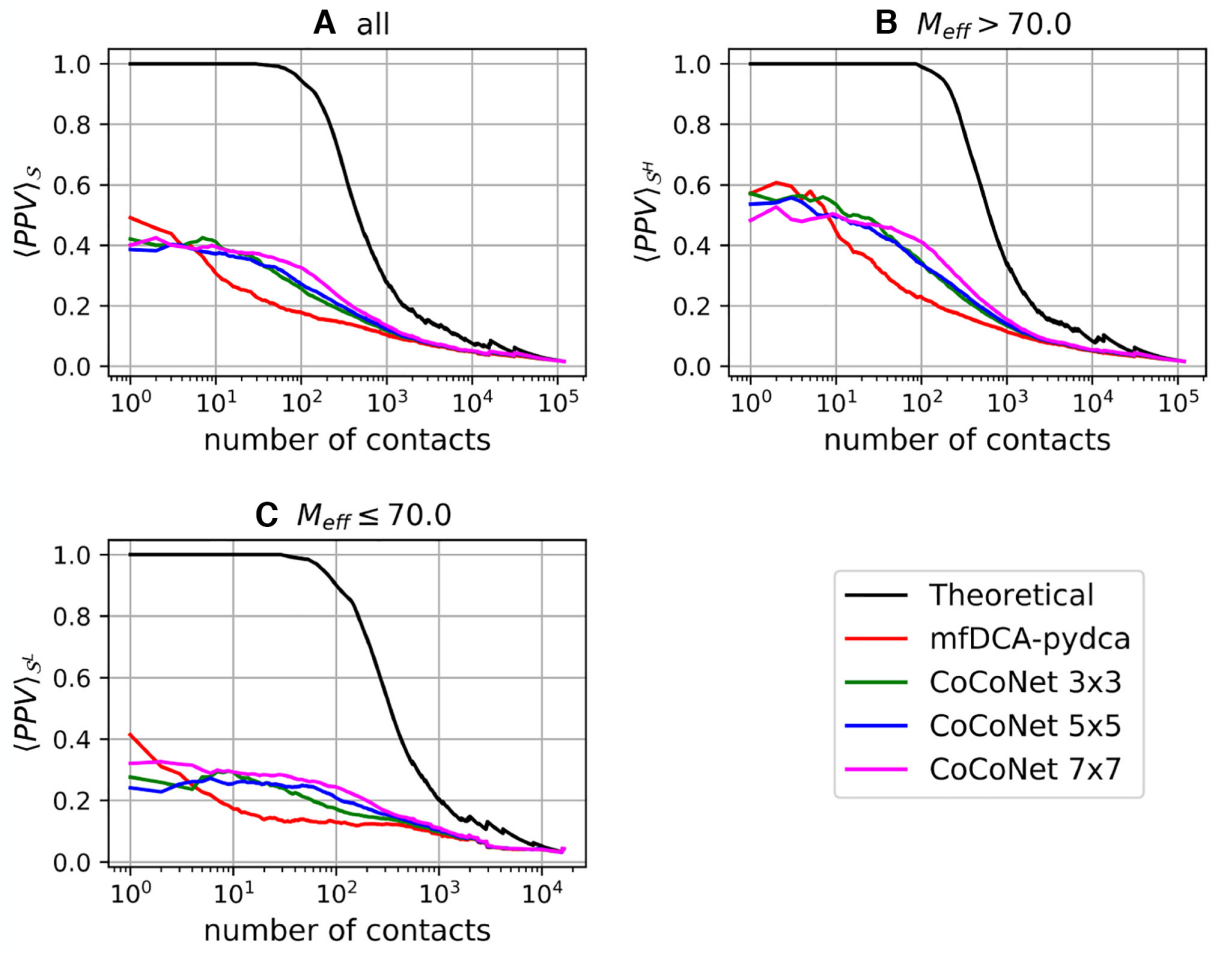
Average positive predicted value of tertiary contacts as a function of the number of contacts for all families in the $\mathcal {S}$ (**A**), $\mathcal {S}^{H}$ (**B**) and $\mathcal {S}^{L}$ (**C**) datasets. CoCoNet’s results are obtained by 5-fold cross-validation.

Figures 3 and 4 show the average PPVs as a function of rank for all pairs (*i*, *j*) such that |*i* − *j*| > 4 (see Supplementary Figure S1 in the supplementary material for individual RNA’s PPV) and that of tertiary contacts, respectively. Nucleotide pairs are considered as tertiary contacts if they are not secondary structure pairs (in particular, secondary structure does not include pseudoknot pairs) and are not in a 5 × 5 window around 2D contacts.

In both cases, CoCoNet shows a significant increment of PPVs for almost all ranks over mean field DCA (30). From the comparison of CoCoNet with other state-of-the-art DCA algorithms such as the Boltzmann learning (31), two implementations of the pseudo-likelihood maximization (EVCoupling (29) and pydca (30)) and graphical lasso (PSICOV) (27) shown in Supplementary Figures S2, we found the same trend with substantially improved CoCoNet PPV values with respect to the other methods. These results clearly indicate the ability of the convolutional layer to improve contact prediction accuracy via our shallow neural network.

Although no significant difference can be observed at higher ranks (for top ∼5/10 nucleotide pairs) between DCA-based method and CoCoNet, the predictive capacity of CoCoNet is superior to mfDCA if more lower ranks are taking into account. Among the different filter sizes, the 3 × 3 filter matrix performs slightly better than other filter matrices up to ranks of about hundred and slightly worse beyond that limit.

The performance of our method depend, as expected, on the effective number of homologous RNA sequences in the corresponding RFAM family of the target RNA. For families with *M*_*eff*_ > 70 the average PPVs are significantly better than those of families that have lower effective number of sequences (*M*_*eff*_ ≤ 70). This trend is consistent for both classes of contacts, i.e., all and tertiary contacts as we can see in Figures 3 and 4, respectively. Nevertheless, CoCoNet outperforms mfDCA in both scenarios.

We also report the CoCoNet numerical results in Table 1 where the average PPVs for top *L* contacts are displayed for different network characteristics. When all contacts are considered, the performances of mean-field DCA that shows an average PPV of 45% are drastically increased to 74.5% and 77% for single and double filter versions of CoCoNet, respectively. No filter-size dependence is observed here but a slight improvement occurs by using double filter convolution with respect to the single filter ones.

**Table 1.** Average positive predicted value (〈*PPV*〉) for all RNAs in the $\mathcal {S}$ dataset. The first two columns indicates the number and size of filter matrices used, respectively. The third column correspond to the number of free parameters to learn. The fourth and last columns show 〈*PPV*〉 at rank *L* for all and tertiary contacts, respectively. The bottom rows show the 〈*PPV*〉s computed using state-of-the-art DCA algorithms mean-field DCA, two different implementations of the pseudo-likelihood maximization (plmDCA-EVC and plmDCA-pydca), Boltzmann learning and graphical LASSO algorithm (PSICOV)

CoCoNet				
Filter	Filter	Free	〈PPV〉_ALL_	〈PPV〉_3D_
	Size	Param.	(top *L*)	(top *L*)
1	3x3	9	74.6	27.1
1	5x5	25	74.6	29.2
1	7x7	49	74.4	33.6
2	3x3	18	76.5	26.6
2	5x5	50	77.7	27.1
2	7x7	98	77.3	35.0
Mean-field DCA			45.0	17.7
plmDCA-EVC			46.6	16.8
plmDCA-pydca			45.0	16.2
Boltzmann learning			45.7	17.3
PSICOV			39.9	15.0

Tertiary contact prediction capability is also significantly improved by our method (see Table 1) despite the fact that their coevolutionary signals are weaker than 2D contacts as observed in the previous subsection. We note here a dependence on filter matrix size since its increment is reflected by a mild increases of the PPVs (see Table 1, last column). Still, all approaches of CoCoNet outperform standard mfDCA by a large margin, e.g. 35.0% versus 17.7% when using double 7 × 7 filter matrix convolution.

We also list in Table 2 the average PPVs at rank *L* for the two subsets $\mathcal {S}^{L}$ and $\mathcal {S}^{H}$ observing a strong improvement of the CoCoNet performances in both sets: considering all contacts in $\mathcal {S}^{H}$ CoCoNet reaches an average PPV of about 90% in comparison with 57.1% obtained from mean-field DCA. For the dataset $\mathcal {S}^{L}$, CoCoNet’s results are even, surprisingly, higher reaching PPVs between 60% and 67% in comparison with 33% obtained from mean-field DCA. Similar trends are observed for tertiary contacts that are predicted with less accuracy even if their prediction remains significantly improved in both sets (see Table 2).

**Table 2.** Average positive predicted values 〈*PPV*〉 for for all RNAs in the $\mathcal {S}$ dataset. The first two columns indicates the number and size of filter matrices used, respectively. The third and fourth columns show 〈*PPV*〉 at rank *L* in the $\mathcal {S}^H$ dataset for all and tertiary contacts, respectively. Finally, the fifth and sixth columns show 〈*PPV*〉 at rank *L* in the $\mathcal {S}^L$ set for all and tertiary contacts, respectively.The bottom rows show the 〈*PPV*〉s computed using the state-of-the-art DCA algorithms mean-field DCA, two different implementations of the pseudo-likelihood maximization (plmDCA-EVC and plmDCA-pydca), Boltzmann learning and graphical LASSO algorithm (PSICOV)

CoCoNet					
Filter	Filter	〈PPV〉_ALL_	〈PPV〉_3D_	〈PPV〉_ALL_	〈PPV〉_3D_
	Size	$\mathcal {S}^H$	$\mathcal {S}^H$	$\mathcal {S}^L$	$\mathcal {S}^L$
1	3 × 3	90.3	35.0	59.4	19.5
1	5 × 5	87.4	35.0	62.3	23.8
1	7 × 7	86.3	40.3	62.8	27.1
2	3 × 3	91.7	34.7	61.8	18.8
2	5 × 5	89.6	32.0	66.1	22.4
2	7 × 7	87.7	40.3	67.2	29.8
Mean-field DCA		57.1	22.0	33.3	13.6
plmDCA-EVC		61.3	22.0	32.5	11.9
plmDCA-pydca		57.6	22.0	33.0	10.6
Boltzmann learning		58.7	22.2	33.1	12.5
PSICOV		50.9	18.6	29.1	11.5

Finally, we compute the performances of CoCoNet on the independent test set $\mathcal {T}$ containing crystallographic structures with low resolution or NMR structures. Results are reported in Tables 3 and 4 and confirm the significant improvement of the CoCoNet performances for all values of filter number and size with respect to mean-field DCA. Note that these results suggest a slightly better performance for small filters with an improved averaged PPV of almost 90 % with respect to pure DCA-based computation.

**Table 3.** All contact types average positive predictive value (〈*PPV*〉) for all RNAs in the independent dataset $\mathcal {T}$. The first two columns indicates the number and size of filter matrices used, respectively. The third, fourth and fifth columns correspond to the 〈*PPV*〉 at rank *L* for all RNA structure belonging $\mathcal {T}$ and to its subset $\mathcal {T}^H$ and $\mathcal {T}^L$ respectively. The bottom row shows the corresponding 〈*PPV*〉s for DCA-based algorithms

CoCoNet				
Filter	Filter	〈PPV$\rangle _{\mathcal {T}}$	〈PPV$\rangle _{\mathcal {T}^{H}}$	〈PPV$\rangle _{\mathcal {T}^{L}}$
	Size	(top *L*)	(top *L*)	(top *L*)
1	3 × 3	61.2	84.5	51.0
1	5 × 5	59.9	78.3	51.8
1	7 × 7	60.4	79.6	52.0
2	3 × 3	65.1	84.6	56.6
2	5 × 5	64.6	78.6	58.5
2	7 × 7	65.4	80.1	59.0
Mean-field DCA		37.4	54.8	29.7
plmDCA-EVC		35.3	53.6	27.3
plmDCA-pydca		32.9	48.2	26.3
Boltzmann learning		33.9	49.9	26.9
PSICOV		30.7	42.3	25.6

**Table 4.** Tertiary contacts average positive predictive value (〈*PPV*〉) for all RNAs in the independent dataset $\mathcal {T}$. The first two columns indicates the number and size of filter matrices used, respectively. The third, fourth and fifth columns correspond to the 〈*PPV*〉 at rank *L* for all RNA structure belonging $\mathcal {T}$ and to its subset $\mathcal {T}^H$ and $\mathcal {T}^L$ respectively. The bottom five rows show the corresponding 〈*PPV*〉s for DCA-based algorithms

CoCoNet				
Filter	Filter	〈PPV$\rangle _{\mathcal {T}}$	〈PPV$\rangle _{\mathcal {T}^{H}}$	〈PPV$\rangle _{\mathcal {T}^{L}}$
	Size	(top *L* 3D)	(top *L* 3D)	(top *L* 3D)
1	3 × 3	26.0	47.8	16.5
1	5 × 5	25.1	46.8	15.7
1	7 × 7	26.3	47.9	16.9
2	3 × 3	26.0	45.4	17.5
2	5 × 5	24.5	42.8	16.5
2	7 × 7	28.3	50.9	18.4
Mean-field DCA		14.4	26.8	9.0
plmDCA-EVC		13.2	25.8	7.7
plmDCA-pydca		13.7	25.2	8.7
Boltzmann learning		12.7	25.5	7.1
PSICOV		11.6	20.6	7.6

### An example of CoCoNet application

**Figure 5.**
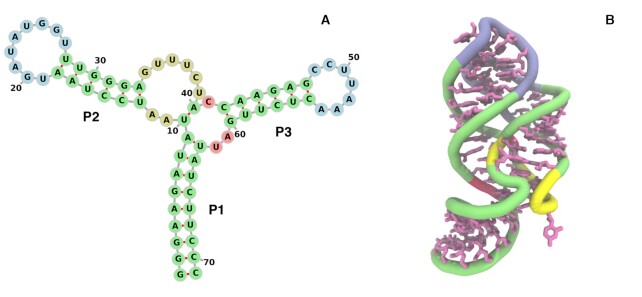
(**A**) Secondary and (**B**) tertiary structure of the *Vibrio Vulnificus* Adenine Riboswitch. The secondary structure diagram was done using (59).

To provide an example of the CoCoNet application we consider the aptamer domain of the Adenine Riboswitch from *Vibrio vulnificus* that has a known experimentally resolved 3D structure (see figure 5, PDB code 4TZX) (58). This riboswitch is located in the 5’ untranslated region of the *add* adenosine deaminase mRNA and plays an important role in the translational machinery. If the adenine concentrations is high enough, the aptamer domain can bind to the adenine, induce an allosteric conformational change in the binding domains and initiate the translation. The structure consist of a three-way junction connecting three helices P1, P2 and P3 (see Figure 5 with long-range three dimensional contacts formed between P2 and P3 to stabilize the 3D structure.

**Figure 6.**
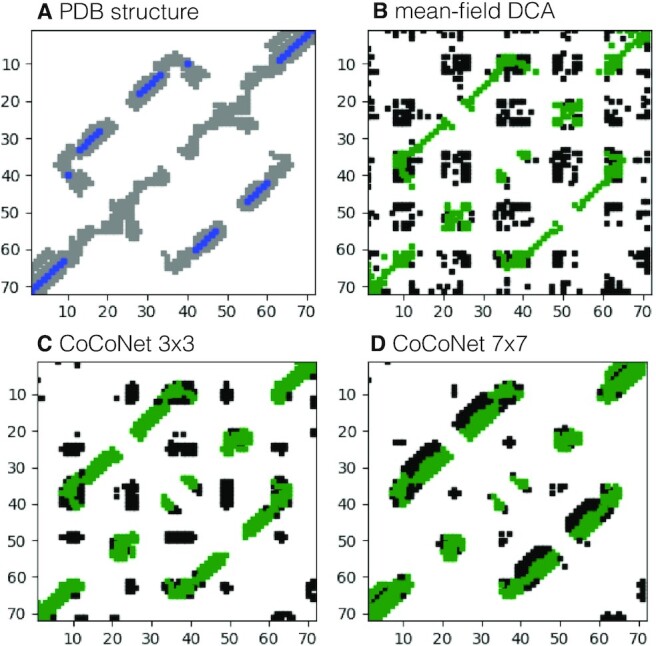
Predicted and experimental contact maps for Adenine Riboswitch from *Vibrio vulnificus* (PDB 4TZX, RFAM RF00167). (**A**) Contacts in the experimentally resolved PDB structure using a heavy atom pair cut-off distance of 10 Å with secondary structure pairs in blue color. (**B**) Mean-field DCA predicted contact map with true/false positives highlighted in green/black. (**C**,**D**) CoCoNet predicted contact map using single 3 × 3 and 7 × 7 filter matrix respectively with the green/black color indicating true/false positives.

The experimental contact map of this Riboswitch is displayed in Figure 6A where we highlight the nucleotide pairs having at least a pair of heavy atoms less than 10 Å apart. Among all these 382 contacts, the secondary structure pairs are colored in blue whereas the remaining contacts are colored in grey. Figure 6 B display the contact map constructed by taking the top 382 mean-field DCA predicted nucleotide pairs: 38% of them are true positives (colored in green) and the rest are false positives (colored in black). Finally, Figure 6C and D represented CoCoNet predicted top 382 nucleotide pairs using 3 × 3 and 7 × 7 single filter convolution, respectively. As we can clearly see from this picture, CoCoNet (with a PVV of 60% and 67% for 3 × 3 and 7 × 7 filter size, respectively) improves the performances of mfDCA (PPV equal to 38%) substantially.

These contact maps clearly show the ability of CoCoNet to significantly enhance contact prediction from coevolutionary signals initially identified by DCA. The mfDCA contact map has indeed false positives scattered all over the contact map. When convolution is performed on top of coevolution, false positives are suppressed while true positives are enhanced and tend to cluster around strongly coevolving pairs. Finally, in Figure 6C and D we can also see that the clustering power of CoCoNet is enhanced for large filter matrix size as already observed previously when the number of contacts considered is large enough.

### RNA 3D structure prediction

In this section, we test how the predicted contacts affect the 3D RNA structure prediction. For the structural modeling, we used SimRNA (9). As there is no well-established way for RNA to include contact constraints, we include contacts in the simulations in three different ways: in the first case, only the top *L* contacts are considered; in the second one, the top 2*L* and in the third one only the top *L* tertiary contacts. The latter case demands special care, as a priori in a true blind prediction, we do not know whether a predicted contact is 2D or 3D. We therefore use the RFAM consensus sequence and its conserved 2D structures to define the 2D contacts even if they differ from the real ones.

The average results and spreads are shown in Table 5 while the values for each RNA are reported in Supplementary Table S3. To our surprise, despite the impressive increase of PPV performances, CoCoNet contact predictions do not improve 3D modeling results beyond the results for mfDCA contacts. From the set of 10 predicted RNA structures, in 4 cases, CoCoNet performs better than mfDCA with a lower RMSD between predicted and experimental structures, in other 4 RNAs, mfDCA performs better than CoCoNet, in one they reach precisely the same RMSD, while in the last case the SimRNA without any contact performs better than both CoCoNet and mfDCA (see Supplementary Table S3, values in blue).

**Table 5.** Result of the RNA 3D modeling in terms of RMSD (in Å) between the predicted and the experimental 3D structures averaged over the ten RNAs considered in the analysis. The twenty lowest energy clusters for each RNA are considered, and the averaged 〈RMSD〉 as well as the standard deviation σ of their RMSD are reported in the third and fourth column, respectively

	#	〈 RMSD 〉	σ
	Contact	(All)	(All)
SimRNA	-	14.8	5.5
SimRNA+mfDCA	Top *L*	11.7	5.3
SimRNA+mfDCA	Top 2*L*	14.5	3.7
SimRNA+mfDCA	Top 3D *L*	12.8	6.0
SimRNA+PSICOV	Top *L*	11.5	5.0
SimRNA+PSICOV	Top 2*L*	15.0	4.7
SimRNA+PSICOV	Top 3D *L*	17.1	4.4
SimRNA+Boltzmann learning	Top *L*	12.6	4.3
SimRNA+Boltzmann learning	Top 2*L*	12.9	4.4
SimRNA+Boltzmann learning	Top 3D *L*	16.4	4.4
SimRNA+CoCoNet_5 × 5_	Top *L*	12.6	4.5
SimRNA+CoCoNet_5 × 5_	Top 2*L*	13.4	4.0
SimRNA+CoCoNet_5 × 5_	Top 3D *L*	13.4	4.5

## DISCUSSION

The accurate prediction of nucleotide-nucleotide contacts in RNA molecules remains an intriguing and challenging issue whose resolution could boost RNA structure prediction and shed light on fundamental properties of RNA and on its functions within the cell. Unfortunately, the limited number of resolved RNA structure prevents the application of complex machine learning models coupled or not with coevolutionary-based methods that recently have been successfully applied to proteins (34,35).

In this paper, we made a significant improvement in RNA contact prediction circumventing this limitation by using a combination of direct coupling analysis and a very simple convolutional neural network. Although the model has very few parameters, it is able to enhance contact prediction accuracy using limited RNA sequence data. Indeed the CoCoNet averaged PPV for a set of 57 RNAs that belong to distinct families of homologous RNA, improves the results of mean-field DCA with a PPV of 45.0% up to about 77.0% when top *L* ranked nucleotide pairs are considered. Remarkably, we observe that tertiary contact prediction is significantly improved from a PPV value of about 17.0% for the mean field DCA up to about 33.0%. Analogous results can be found in the comparison of CoCoNet with other known DCA-based algorithms such as Boltzman learning and pseudo-likelihood maximization DCA.

Finally, the same highly significant improvements of the performances are observed when we tested CoCoNet on an independent test set of 23 RNA structures that were not included in the training procedure.

These improvements are achieved by performing convolution operation on top of coevolution and thus learning patterns of coevolving nucleotide pairs using simple filter matrices. The enhancement effect can be observed for either strong co-evolutionary signals but also for weaker ones that in principle are more easily confused with the background noise, as in the case of the 3D contacts or in the case of the homologous families with a limited number of RNA sequences.

However, despite the impressive increase of PPV performances, the straightforward inclusion of predicted CoCoNet contacts in the 3D modeling does not improve the 3D structure prediction accuracy with respect to mfDCA. Our analysis of the results suggests that not all the additional predicted contacts seem to carry valuable information for structural modeling. PPV, by construction, only measures contact reliability without considering the context of the entire predicted contact map. As we trained CoCoNet performance on this metric, which is the literature standard to compare contact prediction performance, it succeeds in optimizing PPV but this does not translate in the additional desired property of also directly and reliably providing improved structure prediction constraints. Here, either a better metric or improved constraint integration in 3D modeling tools seems necessary. Such a better metric is not trivial, as it would have to (A) weight each predicted contact in its assumed benefit for 3D modeling ideally in (B) the context of the other predicted contacts. (A) could be realized, for example, by weighting contacts with measures such as contact order from the protein folding field (60). (B), however, is likely more difficult to achieve. One could image weighting the distribution diversity of the contact map with clusters of contacts being less valuable.

We can therefore explore multiple directions to further improve our method to better understand the structural properties of RNA molecules. First of all, when more 3D RNA structures will be experimentally available we could exploit more complex neural networks architecture to improve the accuracy of our method. In addition, although CoCoNet is able to enhance RNA tertiary contact prediction, their prediction accuracy remains limited and thus needs to be further improved. This is a challenging issue since as we have seen in previous sections the co-evolutionary signals are dominated by the secondary structures. Also, we believe that with a dedicated analysis, we could find an alternative way to integrate CoCoNet constraints in molecular modeling tools and improve and boost the structural RNA models’ accuracy. Additionally, as discussed in the last paragraph, we would suggest discussion in the community to identify a robust quantitative measure of contact map prediction quality beyond PPV, which also takes into account the information content improvement for 3d structural modeling of RNA.

## Supplementary Material

gkab1144_Supplemental_File
