# Supplementary material for: CoCoNet—boosting RNA contact prediction by convolutional neural networks
Source: Nucleic Acids Res. 2021 Dec 6;49(22):12661–72. doi: 10.1093/nar/gkab1144 (PMC8682773; doi:10.1093/nar/gkab1144)
Supplement: gkab1144_Supplemental_File [file gkab1144_supplemental_file.pdf]

Supplementary material for: CoCoNet—Boosting RNA contact prediction with convolutional neural network

Mehari B. Zerihun[1,2,*], Fabrizio Pucci[1,3,*] and Alexander Schug[1,4,†]

[1]John von Neumann Institute for Computing, Jülich Supercomputing Centre, Forschungszentrum Jülich, 52428 Jülich, Germany
[2]Steinbuch Centre for Computing, Karlsruhe Institute of Technology, 76344 Eggenstein-Leopoldshafen, Germany
[3]Computational Biology and Bioinformatics, Université Libre de Bruxelles, 1050 Brussels, Belgium
[4]Faculty of Biology, University of Duisburg-Essen, 45117 Essen, Germany
† To whom the correspondence should be addressed: al.schug@fz-juelich.de
∗ These authors contributed equally to this work

October 25, 2021

1. The RNAs in the datasets and their description
2. Individual RNAs positive predictive values
3. Comparing CoCoNet with variants of DCA algorithms
4. Comparing CoCoNet and DCA using the Mathews correlation coefficient
5. RNA 3D modeling
6. CoConet Filter Matrices

1 The RNAs in the datasets and their description

Table S1 lists the RNAs in the dataset $\mathcal{S}$. The Table contains the family names in column two, PDB IDs in column three, the effective number of sequences (M_{eff}) in column four, the length of RNA sequences in column five, the number of all contact types in column six, and tertiary only contacts in column seven.

Table S1: The dataset of RNA annotated with the family name in Rfam database, PDB ID, number of effective sequences (M_{eff}), length, and number of contacts (all contacts and tertiary only contacts).

	Family Name	PDB ID	M_{eff}	Length	All Contacts	Tertiary Contacts
1	RF00100	5lys	1679.745	57	254	88
2	RF00168	3dil	1455.511	174	1036	556
3	RF00010	1u9s	1285.282	155	854	466
4	RF00059	3d2g	1241.931	77	417	243
5	RF00379	4qln	998.938	117	549	300
6	RF00504	3ox0	847.594	87	442	209
7	RF00028	1gid	799.302	158	951	513
8	RF00234	2h0s	603.709	125	687	484
9	RF01051	4yaz	583.357	84	429	215
10	RF00005	1ehz	461.009	76	370	190
11	RF00167	4tzx	458.874	71	382	199
12	RF00080	6cb3	409.471	101	509	243
13	RF01786	3q3z	372.130	75	363	222
14	RF00162	3gx5	322.101	94	509	273
15	RF00169	1z43	281.462	101	512	248
16	RF01852	3rg5	268.101	86	398	176
17	RF02001	4y1o	248.274	258	1516	878
18	RF01734	4enc	222.076	52	209	128
19	RF01725	4l81	210.969	96	483	277
20	RF01831	4lvv	187.739	89	486	269
21	RF01854	4wfl	176.994	107	562	323
22	RF00011	1nbs	157.580	120	662	414
23	RF01750	4xwf	130.655	64	232	85
24	RF02540	1ffz	120.116	497	3139	1937
25	RF00380	3pdr	119.645	161	1088	663
26	RF02683	4rum	117.035	93	406	155
27	RF00050	3f2q	104.843	108	650	405
28	RF02553	6cu1	91.370	80	393	180
29	RF01689	4frg	62.800	84	438	246
30	RF00163	3zp8	59.045	43	131	88
31	RF00029	1kxk	50.960	70	252	66
32	RF02682	3nkb	40.976	64	295	159
33	RF00442	5u3g	33.886	85	429	225
34	RF02679	5k7d	32.373	47	203	150
35	RF02695	4k27	32.000	55	210	54
36	RF00233	4p5j	16.221	86	463	275
37	RF01054	4jf2	14.558	77	391	233

38	RF00458	2il9	14.000	135	521	315
39	RF01767	3e5c	13.621	53	212	84
40	RF02447	4jrc	6.771	57	283	143
41	RF01510	3slq	6.643	68	363	178
42	RF01763	5nwq	5.059	41	212	146
43	RF01415	4pqv	4.862	68	296	142
44	RF00061	3t4b	3.609	84	341	139
45	RF00164	1xjr	3.293	47	221	75
46	RF01807	4p95	3.164	189	1145	702
47	RF01826	6fz0	2.806	48	218	156
48	RF02680	4rzd	2.602	99	415	210
49	RF03017	2oiu	2.004	71	224	64
50	RF02927	4r4v	2.000	186	825	326
51	RF00606	5m0h	2.000	42	140	29
52	RF00921	5dun	2.000	54	313	200
53	RF00044	3r4f	2.000	66	213	54
54	RF02681	5t5a	2.000	62	322	150
55	RF02266	4plx	2.000	76	412	287
56	RF01300	5ob3	2.000	69	319	138
57	RF01982	5kpy	2.000	71	346	211

Table S2 lists the RNAs in the dataset $\mathcal{T}$. The Table contains the family names in column two, PDB IDs in column three, the effective number of sequences (M_{eff}) in column four, and the length of the sequence in column five.

Table S2: The dataset of RNA annotated with the family name in Rfam database, PDB ID, number of effective sequences (M_{eff}) and length.

	Family Name	PDB ID	M_{eff}	Length
1	RF00001	1c2x	6901.7	120
2	RF00008	5di2	158.8	48
3	RF00017	1l9a	936.8	128
4	RF00023	1p6v	281.1	68
5	RF00027	5zal	36.1	73
6	RF00102	6ol3	29.7	112
7	RF00166	2mf0	48.2	72
8	RF00174	4gma	7857.4	210
9	RF00207	2ke6	1.2	48
10	RF00209	4c4q	4.0	233
11	RF00210	2nbx	26.7	108
12	RF00374	1s9s	9.9	101
13	RF00480	1z2j	4.4	45
14	RF00500	2krl	2.0	102
15	RF00634	6ues	192.0	119
16	RF01073	2lc8	27.2	63
17	RF01381	2n1q	2.0	155
18	RF01704	6qn3	54.1	50
19	RF01727	6hag	14.2	43
20	RF01739	5ddp	38.2	61
21	RF01857	3ndp	137.0	136
22	RF01998	3bwp	2.6	412
23	RF02012	4r0d	2.0	622

2 Individual RNAs positive predictive values

Figure S1 shows the positive predicted values (PPVs) as a function of the nucleotide pair rank for all RNAs analyzed in this paper. The comparison is between the theoretical values obtained from the experimental RNA structures and the prediction of mfDCA and CoCoNet with convolutional filters of different sizes. The PPVs for CoCoNet are obtained using a strict cross-validation procedure and are averaged over ten trials. In almost all RNAs, CoCoNet shows superior performance over mean-filed DCA.

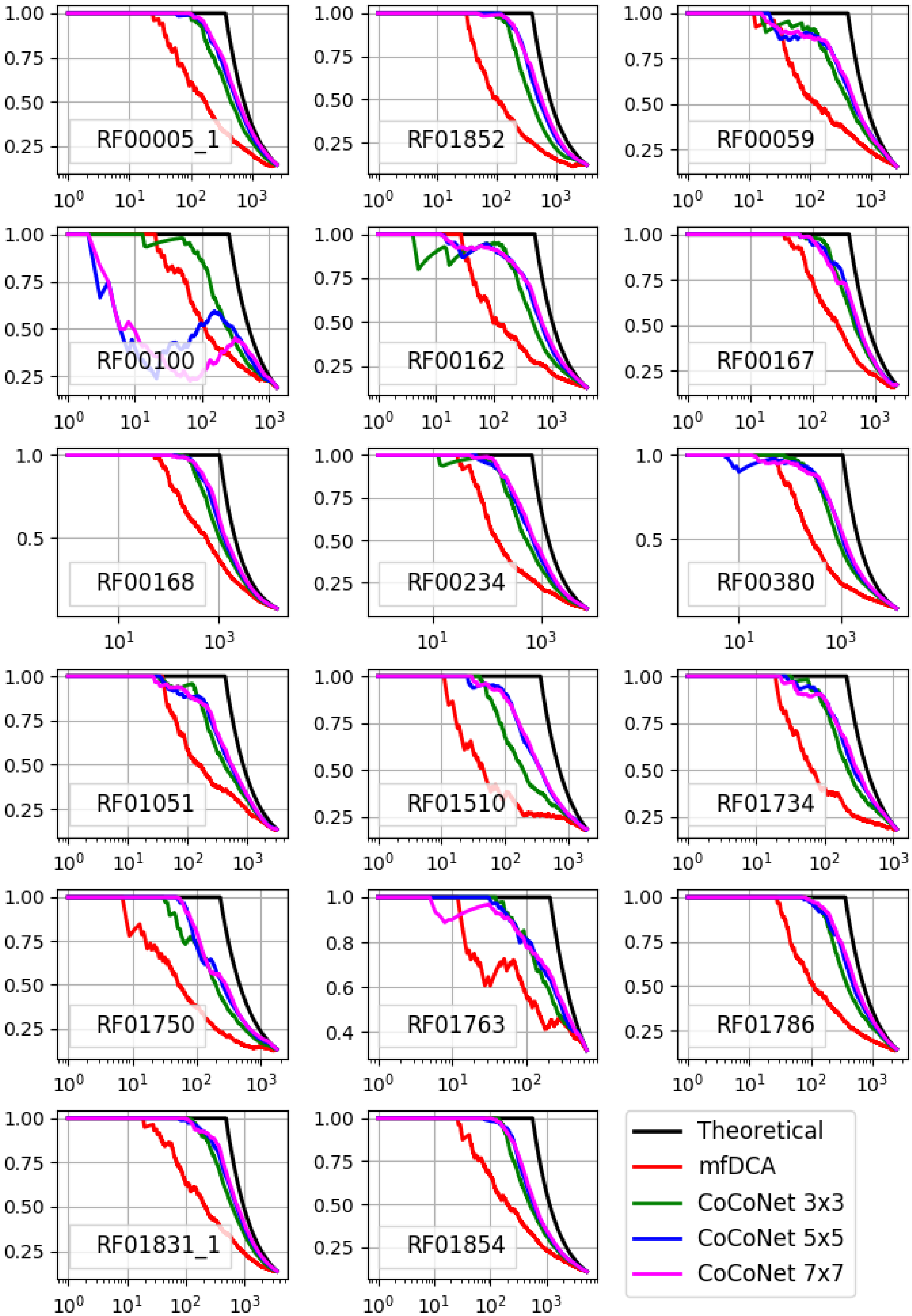
RF00005_1
RF01852
RF00059
RF00100
RF00162
RF00167
RF00168
RF00234
RF00380
RF01051
RF01510
RF01734
RF01750
RF01763
RF01786
RF01831_1
RF01854
Theoretical
mfDCA
CoCoNet 3x3
CoCoNet 5x5
CoCoNet 7x7

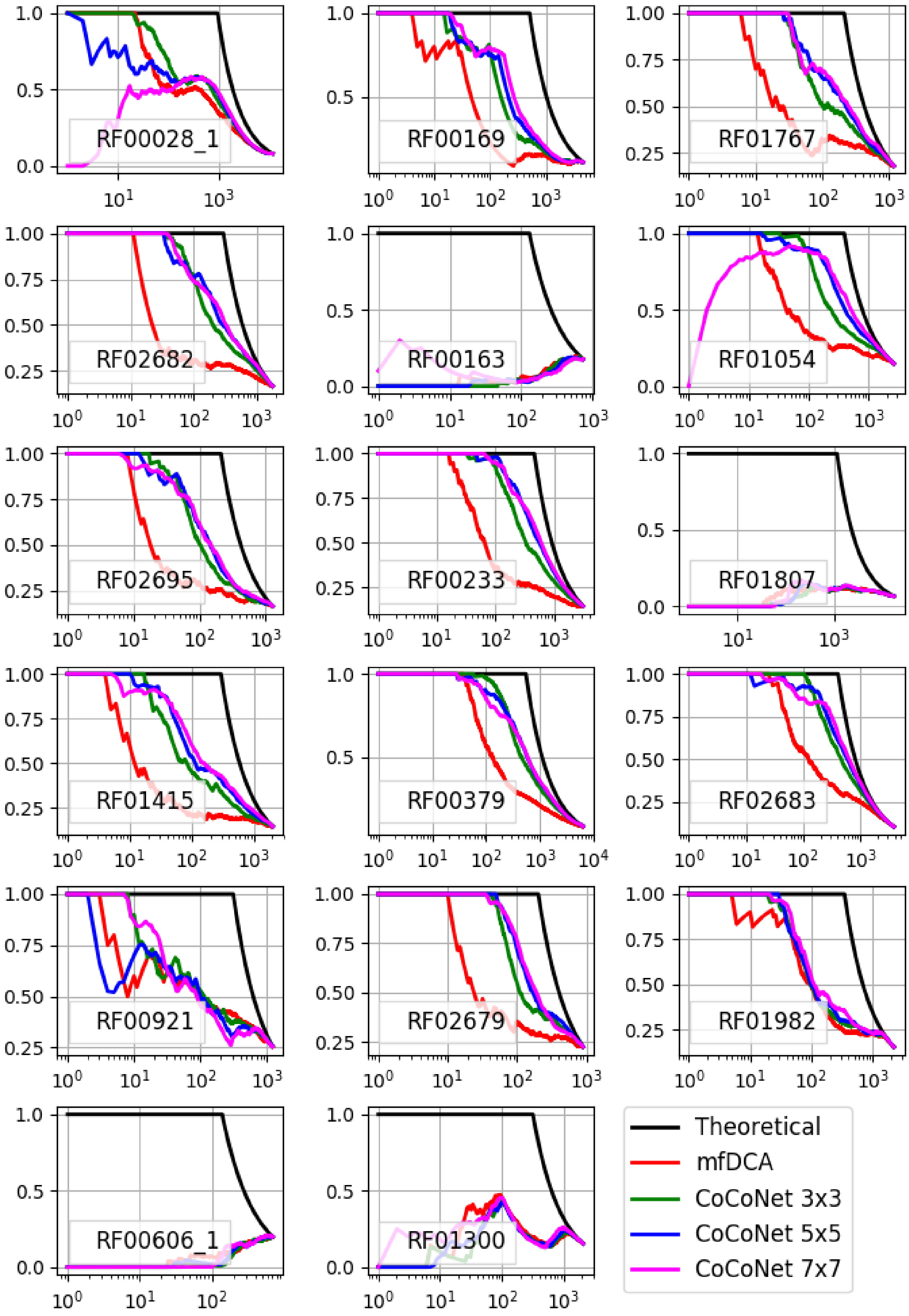

RF00028_1
RF00169
RF01767
RF02682
RF00163
RF01054
RF02695
RF00233
RF01807
RF01415
RF00379
RF02683
RF00921
RF02679
RF01982
RF00606_1
RF01300
Theoretical
mfDCA
CoCoNet 3x3
CoCoNet 5x5
CoCoNet 7x7

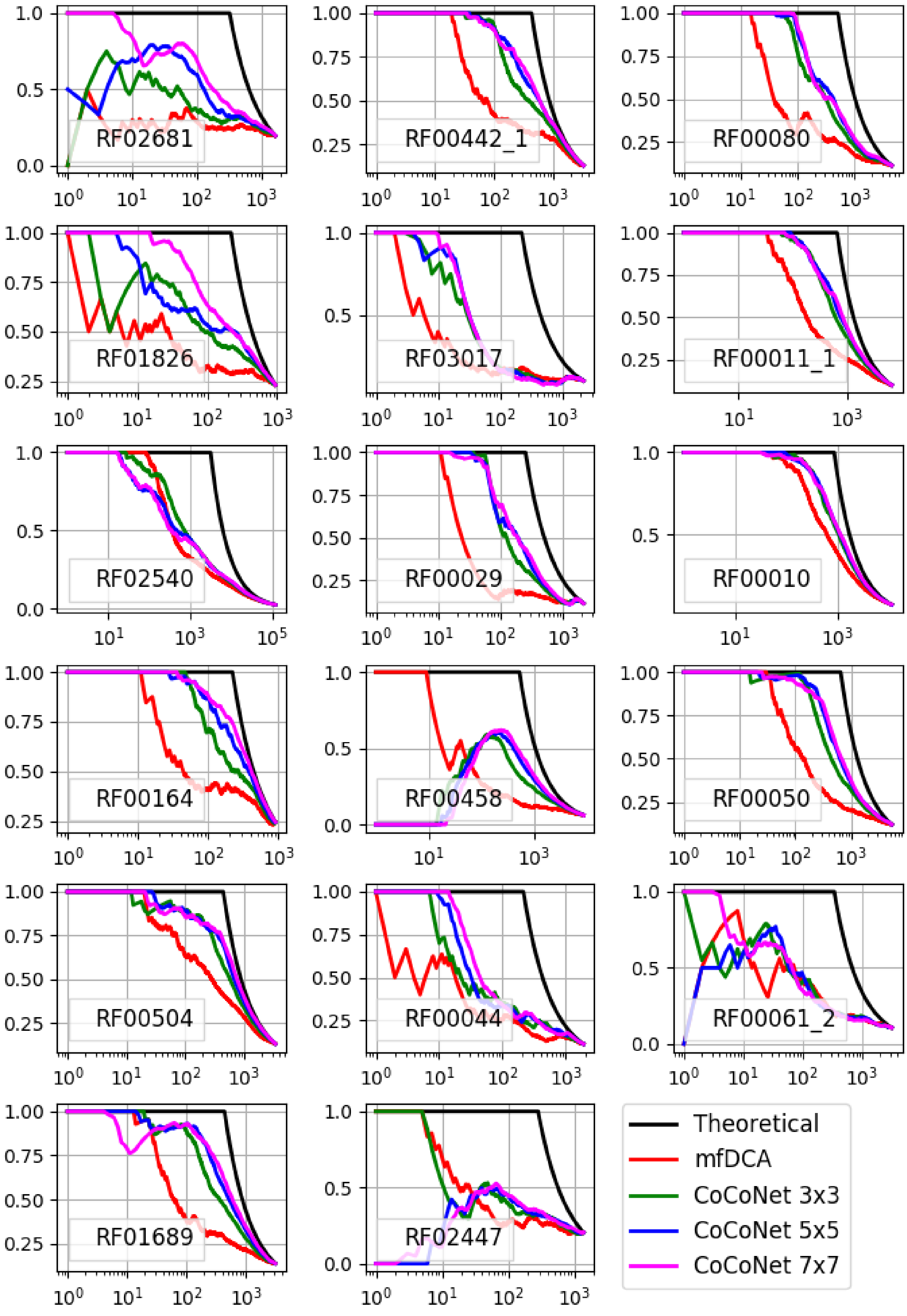
RF02681
RF00442_1
RF00080
RF01826
RF03017
RF00011_1
RF02540
RF00029
RF00010
RF00164
RF00458
RF00050
RF00504
RF00044
RF00061_2
RF01689
RF02447
Theoretical
mfDCA
CoCoNet 3x3
CoCoNet 5x5
CoCoNet 7x7

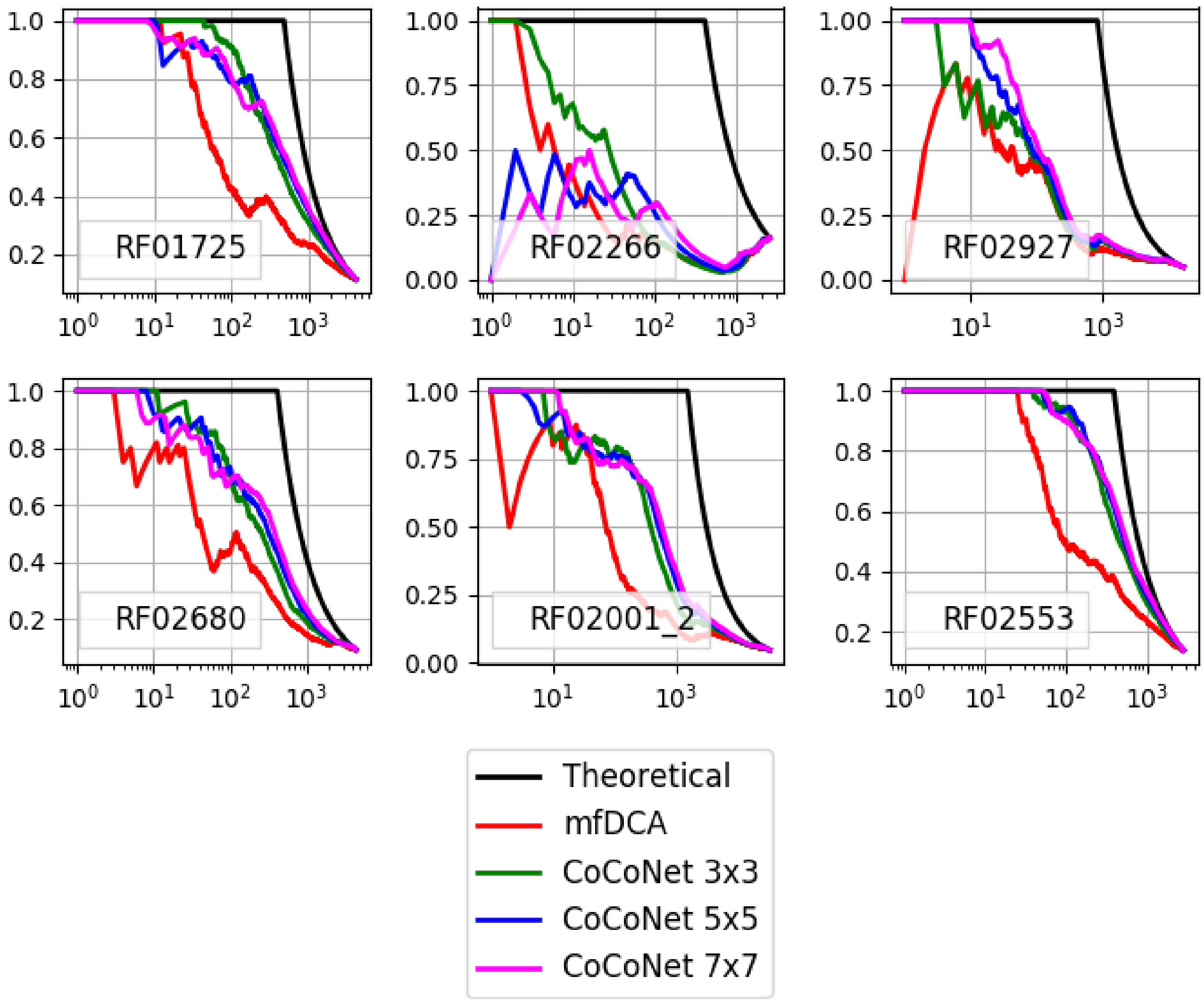

Figure S1: Plots of average PPVs, as a function of the number of contacts for each of the 57 RNAs. The averages are performed over ten trials, each trial containing five-fold cross-validations.

3 Comparing CoCoNet with variants of DCA algorithms

Here, we compare average positive predictive values $\langle PPV \rangle$ of state-of-the-art DCA algorithms with that of **CoCoNet**. Fig. S2 shows the $\langle PPV \rangle$ at full rank.

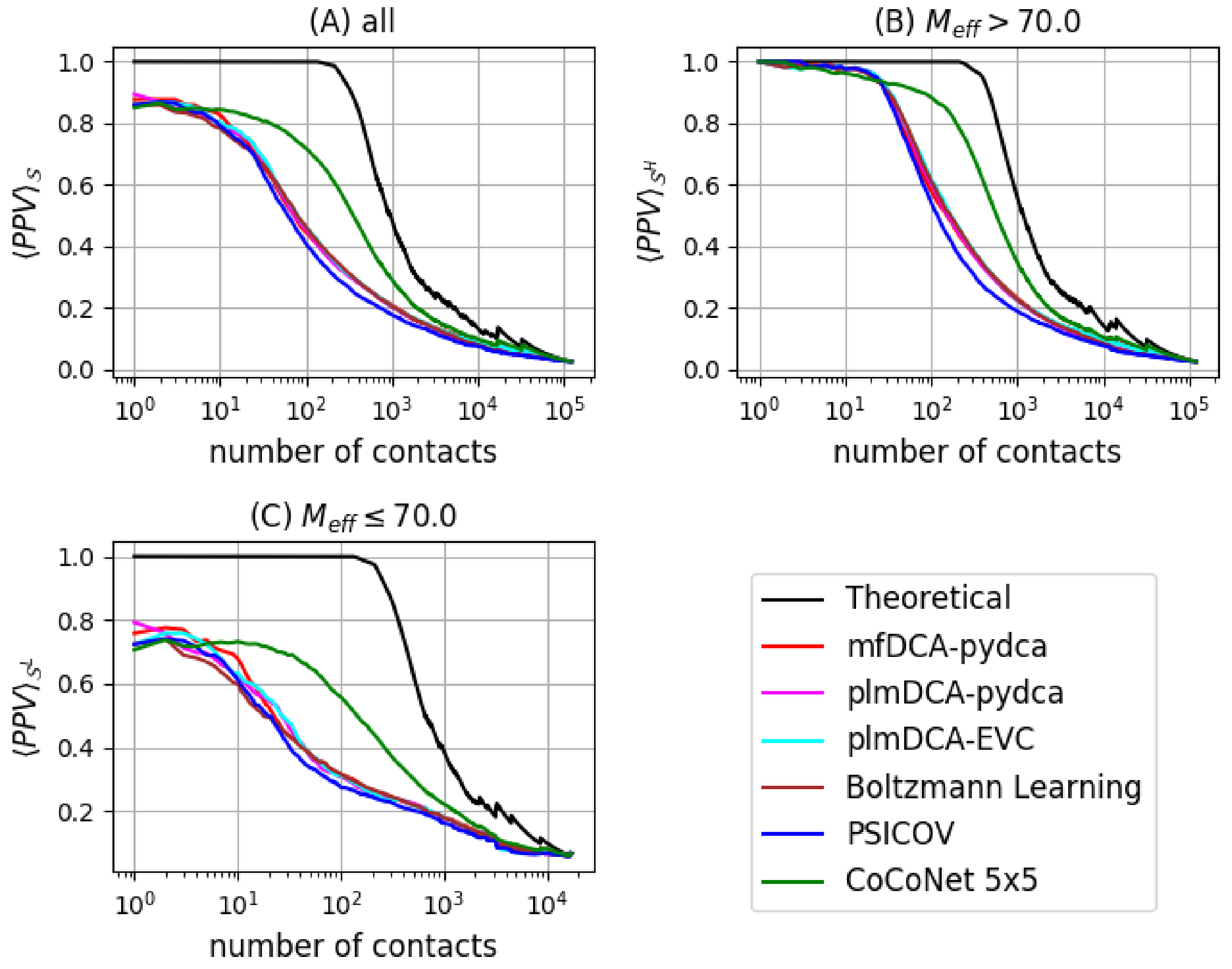

Figure S2: Average positive predictive values as a function of the number of contacts for the DCA algorithms (i) mean-field and pseudo-likelihood maximization in pydca, mfDCA-pydca, and plmDCA-pydca respectively (ii) the pseudo-likelihood maximization of EVCouplings, plmDCA-EVC (iii) Boltzman Learning (iv) the graphical LASSO algorithm implemented in PSICOV and the **CoCoNet** using a 5×5 filter matrix, CoCoNet 5x5. The closer the PPV curve to the Theoretical PPV, the more accurate it is. The subfigures are for datasets $\mathcal{S}$ (top-left Figure); $\mathcal{S}^H$, top-right Figure; and $\mathcal{S}^L$, bottom-left Figure.

4 Comparing CoCoNet and DCA using the Mathews correlation coefficient

We also compare DCA algorithms' performance with **CoCoNet** using the Mathews correlation coefficient (MCC) as a metric. MCC takes into account all elements of the binary confusion matrix when nucleotide pairs are categorized as contacts and non-contacts. The MCC is given by the formula

$$MCC = \frac{(TP)(TN) - (FP)(FN)}{\sqrt{(TP+FP)(TP+FN)(TN+FP)(TN+FN)}}, \tag{1}$$

where TP/FP are the numbers of true/false positive and TN/FN are true/false negatives.

Figs. S3 and S4 display the average MCCs at rank L (sequences' length), for all contact types and tertiary contacts, respectively, computed using various DCA algorithms and **CoCoNet**. In both cases, **CoCoNet** outperforms state-of-the art DCA algorithms.

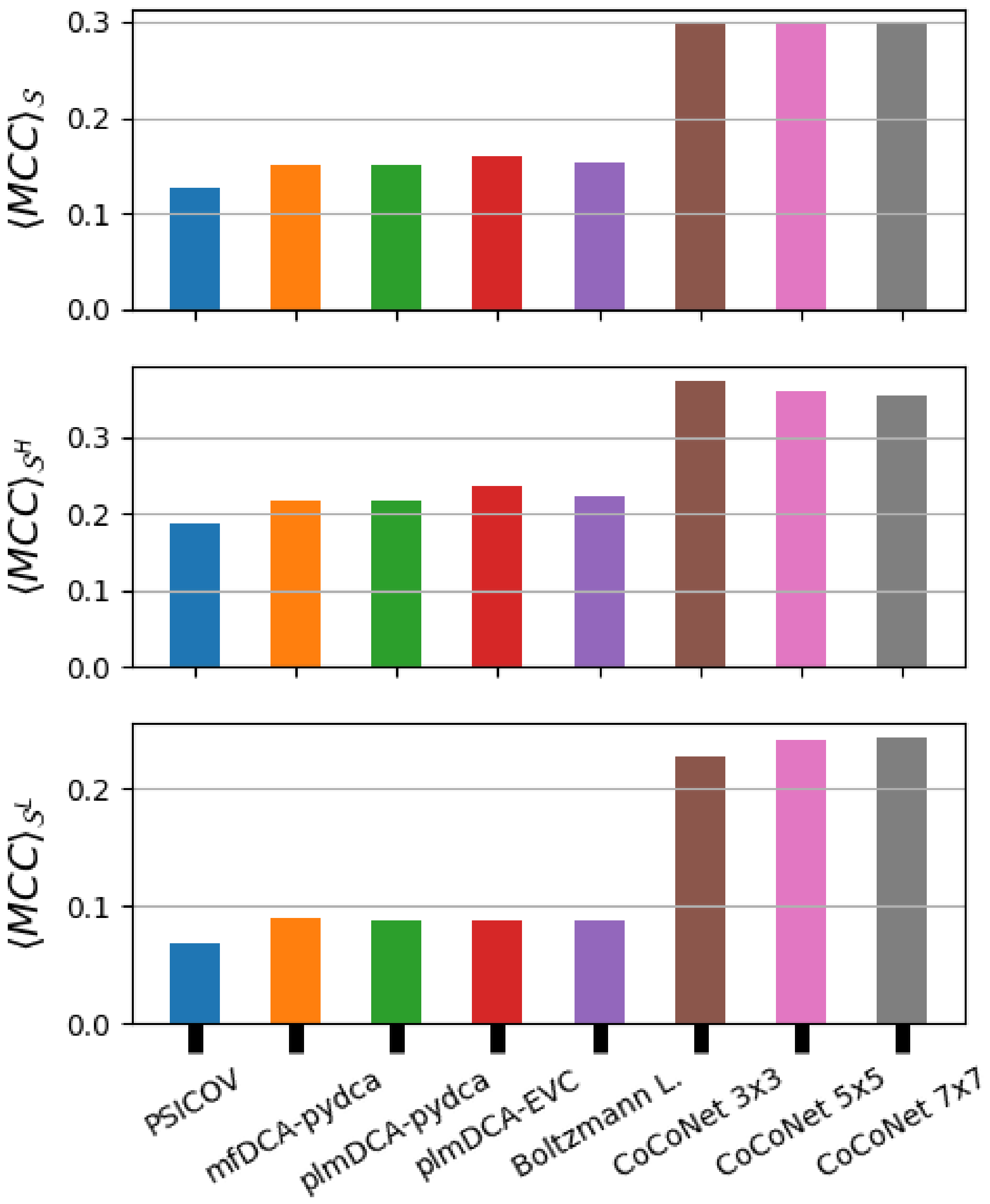

Figure S3: Average MCC, $\langle MCC \rangle$, at rank L for all contact types in dataset $\mathcal{S}$ computed using (i) graphical LASSO algorithms in PSICOV (ii) mean-field and pseudo-likelihood maximization algorithms in pydca, mfDCA-pydca and plmDCA-pydca, respectively (iii) the pseudo-likelihood maximization in EVCouplings, plmDCA-EVC (iv) Boltzmann learning and (v) **CoCoNet** using 3×3, 5×5, and 7×7 filter matrices. **CoCoNet** significantly outperforms all the state-of-the-art DCA algorithms for all RNAs in the dataset $\mathcal{S}$ (top Figure); $\mathcal{S}^H$ (middle Figure) and $\mathcal{S}^L$ (bottom Figure).

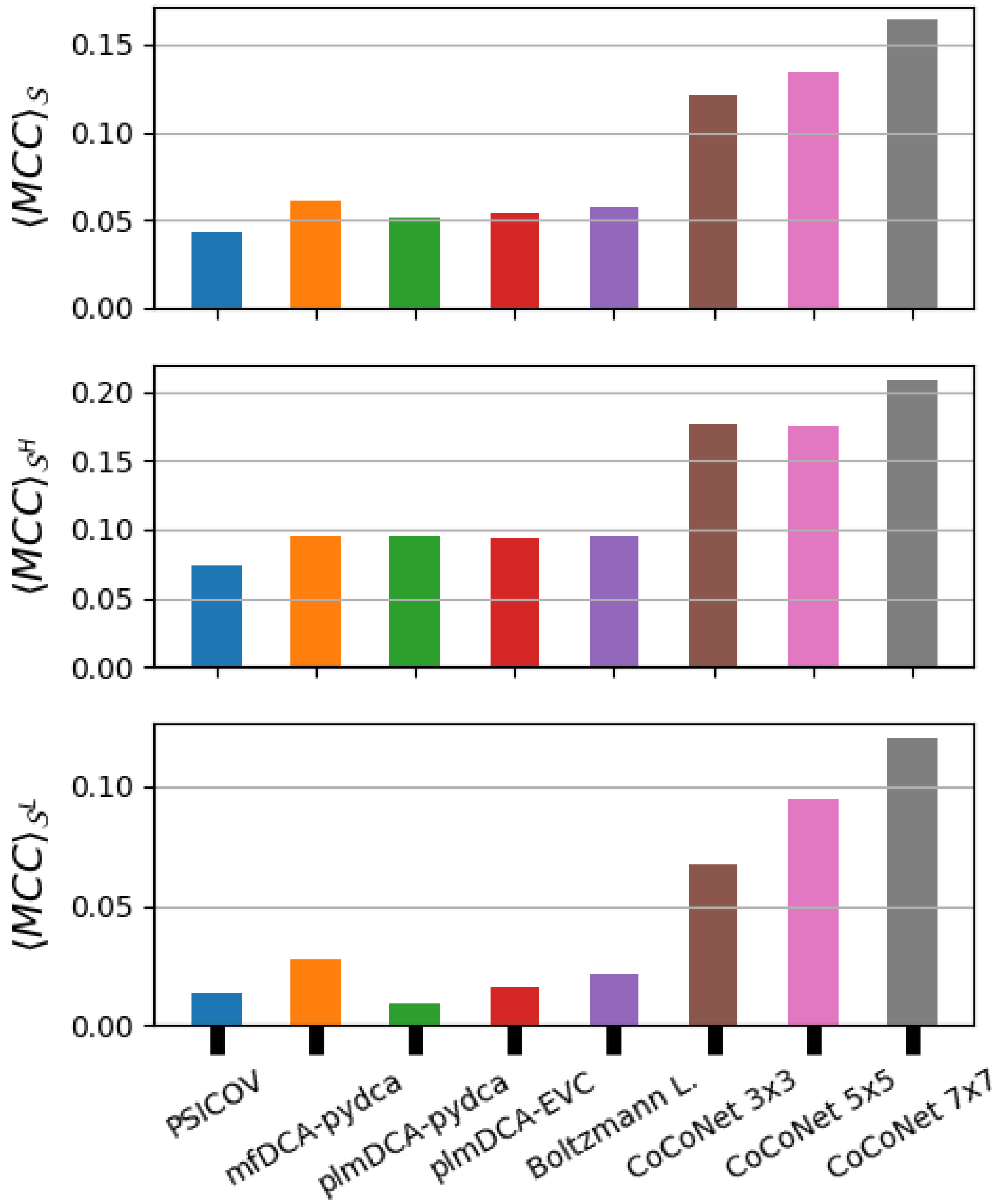

Figure S4: Average MCC, $\langle MCC \rangle$, at rank L for tertiary only contacts in dataset $\mathcal{S}$ computed using (i) graphical LASSO algorithms in PSICOV (ii) mean-field and pseudo-likelihood maximization algorithms in pydca, mfDCA-pydca and plmDCA-pydca, respectively (iii) the pseudo-likelihood maximization in EVCouplings, plmDCA-EVC (iv) Boltzmann learning and (v) **CoCoNet** using 3×3, 5×5, and 7×7 filter matrices. **CoCoNet** significantly outperforms all the state-of-the-art DCA algorithms for all RNAs in the dataset $\mathcal{S}$ (top Figure); $\mathcal{S}^H$ (middle Figure) and $\mathcal{S}^L$ (bottom Figure).

5 RNA 3D modeling

To model the 3D RNA structure, we used the SimRNA tool that is a Monte-Carlo-based approach *(Boniecki et al., Nucleic acids research 44.7 (2016): e63-e63).* For each RNA, we run at least ten times twenty replicas with a temperature between 1.35 and 0.9, with 20×10^6 time steps recording each configuration every 20×10^3 steps. We run four different cases:

- Only the RNA sequence is provided as input
- RNA sequence plus top L contacts from either mean-field DCA or CoCoNet are provided as input
- RNA sequence plus top $2L$ contacts from either mean-field DCA or CoCoNet are provided as input
- RNA sequence plus top L tertiary contacts from either mean-field DCA or CoCoNet are provided as input. Tertiary contacts are defined as usual as contacts that are outside 5×5 windows from any 2D. Since the 2D contacts are not known a priori in a blind prediction, we use here the consensus secondary structure of the RFAM family to define them.

Contacts are included using the "WELL" potentials in SimRNA. Once the configurations are sampled, we chose the one percent configurations with the lowest energies and cluster them with a radius of 3Å. We then selected the first twenty clusters and calculated the RMSD between the experimental PDB structure and the best five, the best ten, and all the 20 structures reporting these values for all the ten RNA structures analyzed in Table S3.

Table S3: Result of the 3D RNA structural modeling. In the first, second, and third columns, we provide the PDB code for all 10 RNAs analyzed in this paper, the corresponding RFAM family, and its number of effective sequences M_{eff}. The fourth, fifth, and sixth columns show the RMSD between the experimental and the predicted RNA structures. The lowest value of each line is highlighted in blue.

PDB	RFAM	M_{eff}		SimRNA	+mfDCA Top ($L/2L$/3D-L contacts)	+CoCoNet 5× 5 Top ($L/2L$/3D-L contacts)
3d2g	RF00059	1241	Best5	15.7 Å	8.5/10.4/18.4 Å	16.2/15.7/16.6 Å
			Best10	17.0 Å	9.8/11.1/18.6 Å	17.8/18.1/17.4 Å
			All	20.0 Å	**10.3**/12.7/19.4 Å	19.6/20.0/19.2 Å
3gx5	RF00162	322	Best5	13.8 Å	12.9/13.3/17.5 Å	13.7/12.4/12.1 Å
			Best10	14.9 Å	13.5/13.7/18.0 Å	14.2/12.7/12.9 Å
			All	18.4 Å	14.4/14.4/20.0 Å	15.1/**13.3**/15.0 Å
3ox0	RF00504	847	Best5	10.4 Å	6.7/14.0/7.4 Å	8.5/5.4/11.0 Å
			Best10	13.1 Å	10.5/14.7/7.8 Å	9.4/6.1/11.6 Å
			All	15.0 Å	13.2/16.0/**8.5** Å	11.2/**8.5**/12.5 Å
4tzx	RF00167	458	Best5	7.5 Å	3.0/10.3/3.2 Å	7.8/13.1/6.6 Å
			Best10	8.1 Å	3.7/13.3/3.7 Å	8.3/14.4/8.3 Å
			All	10.3 Å	5.4/14.4 /**4.2** Å	9.6/15.7/10.1 Å
4yaz	RF01051	583	Best5	16.1 Å	20.1/13.6/16.2 Å	14.0/14.5/14.1 Å
			Best10	17.1 Å	20.5/14.8/17.6 Å	15.5/15.1/14.9 Å
			All	18.9 Å	21.3/16.8/19.0 Å	17.5/**15.9**/16.8 Å
4enc	RF01734	222	Best5	9.7 Å	4.6/8.9/8.9 Å	8.8/9.0/10.7 Å
			Best10	11.5 Å	5.7/10.4/9.3 Å	9.3/9.5/11.2 Å
			All	13.9 Å	**6.6**/12.3/9.8 Å	10.1/10.7/12.0 Å
1kxk	RF00029	51	Best5	4.9 Å	5.8/10.0/6.7 Å	5.9/10.4/4.1 Å
			Best10	5.6 Å	7.0/12.6/8.0 Å	6.4/11.6/5.9 Å
			All	**6.5** Å	9.4/15.3/10.3 Å	7.6/13.2/8.0 Å
3nkb	RF02682	41	Best5	8.8Å	8.2/13.3/10.8 Å	9.2/6.2/7.7 Å
			Best10	9.6 Å	9.8/15.7/12.2 Å	9.6/7.3/10.5 Å
			All	13.1 Å	12.1/18.0/13.7 Å	10.3/**8.8**/14.1 Å
4pqv	RF01415	5	Best5	18.4 Å	15.2/15.9/16.3 Å	15.4/15.2/17.6 Å
			Best10	18.9 Å	15.7/16.2/16.9 Å	16.0/15.5/17.8 Å
			All	21.1 Å	16.7/17.1/17.8Å	16.9/**16.1**/18.1 Å
5nwq	RF01763	5	Best5	9.9 Å	3.3/4.4/4.4 Å	3.4/10.2/6.5 Å
			Best10	10.4 Å	4.0/5.3/4.7Å	5.2/10.7/7.2 Å
			All	11.1 Å	7.0/8.1/**5.2** Å	8.5/11.2/8.4 Å
			Best5	11.5 Å	8.8/11.4/11.0 Å	10.3/11.2/10.7 Å
**Mean**			Best10	12.6 Å	10.0/12.7/11.7 Å	11.2/12.1/11.8 Å
			All	14.8 Å	11.7/14.5/12.8 Å	12.6/13.4/13.4 Å

6 CoCoNet Filter Matrices

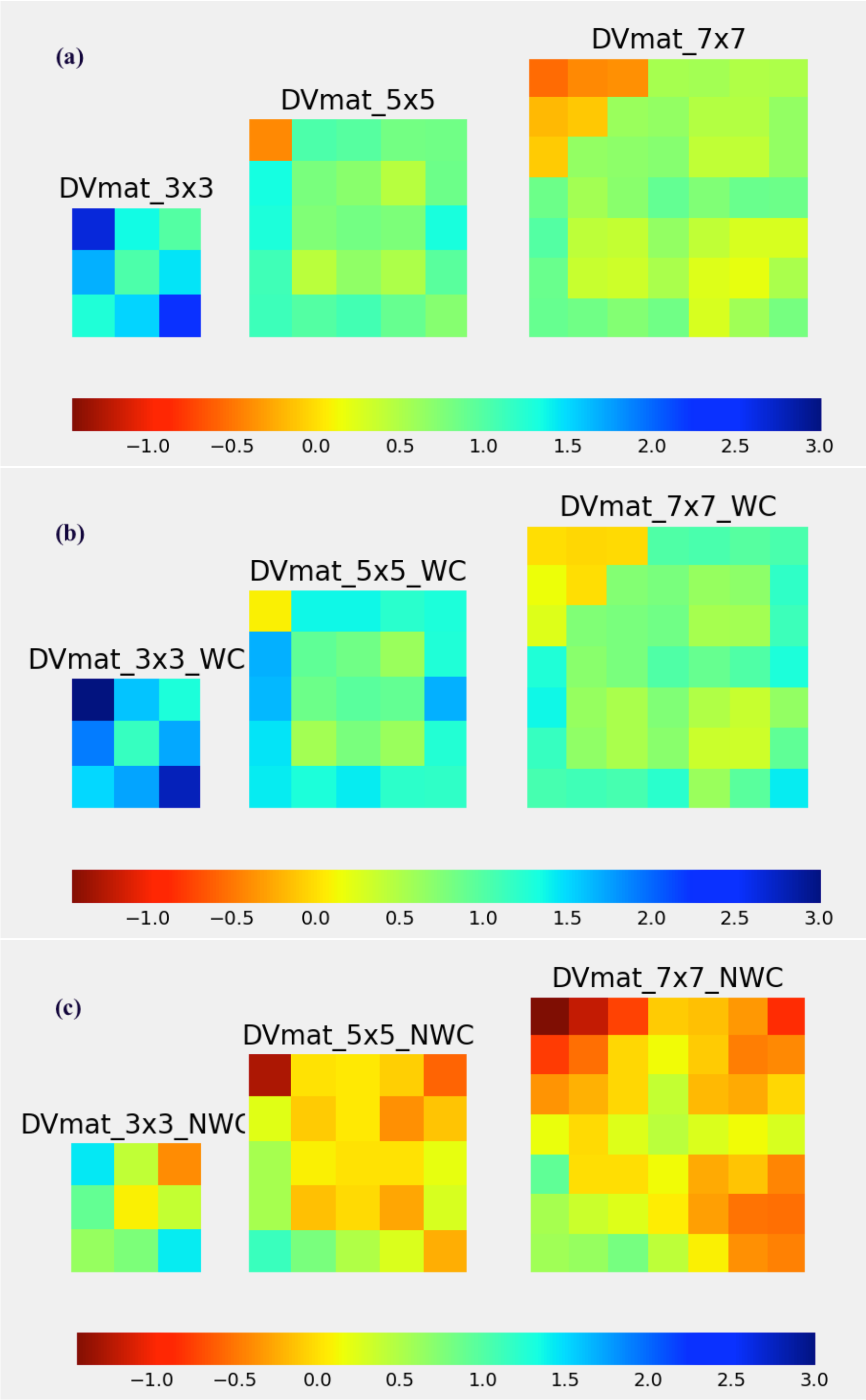

Figure S5: Numerical values of the CoCoNet filters for different filter sizes (3×3, 5×5, 7×7). In (a) there is the standard filter, while in (b) and (c) there are the WC- and non-WC-filters, respectively.
